# EPDM Based Double Slope Triangular Enclosure Solar Collector: A Novel Approach

**DOI:** 10.1155/2014/576101

**Published:** 2014-02-09

**Authors:** Shafiq R. Qureshi, Waqar A. Khan, Waqas Sarwar

**Affiliations:** Pakistan Navy Engineering College, National University of Sciences and Technology (NUST), Karachi 75350, Pakistan

## Abstract

Solar heating is one of the important utilities of solar energy both in domestic and industrial sectors. Evacuated tube heaters are a commonly used technology for domestic water heating. However, increasing cost of copper and nickel has resulted in huge initial cost for these types of heaters. Utilizing solar energy more economically for domestic use requires new concept which has low initial and operating costs together with ease of maintainability. As domestic heating requires only nominal heating temperature to the range of 60–90°C, therefore replacing nickel coated copper pipes with any cheap alternate can drastically reduce the cost of solar heater. We have proposed a new concept which utilizes double slope triangular chamber with EPDM based synthetic rubber pipes. This has reduced the initial and operating costs substantially. A detailed analytical study was carried out to design a novel solar heater. On the basis of analytical design, a prototype was manufactured. Results obtained from the experiments were found to be in good agreement with the analytical study. A maximum error of 10% was recorded at noon. However, results show that error is less than 5% in early and late hours.

## 1. Introduction

Renewable energy is defined as the energy harnessed from natural, rapid, and frequent energy flows occurring in the vicinity of Earth especially on the Earth's surface. Due to the inherent property of balance and reconstruction in nature, the energy extraction from these natural resources is clean, environmently friendly, and has no contribution in the global warming. Solar Energy is a clean and environmently friendly renewable energy resource for sustainable development. Solar energy provides opportunity to reduce dependence on imported fossil fuel by providing an alternative to oil for production of electricity and an alternative to electricity and gas for various domestic, commercial, and industrial heating applications.

Solar water heating for domestic and industrial use is a promising and economical application of solar energy. The temperature requirement of solar water heating systems is generally less than 90°C which is easy to achieve as compared with solar cookers and solar steam generators. Solar water heating finds its utilization in many industries such as leather, beverage, pharmaceutical industry, and textile industry. Swimming Pool and domestic heating requirement are also prominent candidates of solar heating.

There are numerous solar water heating systems used around the world for domestic water heating [1], but the most successful are sheltered tank solar collector, flat plate solar collector, and vacuum tube solar collector. Vacuum tube solar collector is considered the most efficient system for domestic water heating. Flat plate and sheltered tank solar collector systems are more effective for places where the ambient temperature does not fall below 15°C. Although solar collectors have a history extending back to about 120 years ago, the requirements of many diverse applications still continued to be more effectively satisfied with advantages of new materials and manufacturing processes [2].

Evacuated tube solar heaters are fragile and required trained personnel for repair and maintenance. Vacuum tubes themselves are very expensive due to the fact that their use is still limited to specific applications.

This paper aims to present an alternative and cost effective method for domestic heating. This new concept explores the utility of synthetic rubbers instead of metal pipe, which has comparable thermal properties.

## 2. Novel Concept

### 2.1. Basic Concept

New design exploits the concept of green house effect in a glass enclosure. A double slope triangular enclosure is used for trapping the maximum sun radiation in all times of a day.

The triangular enclosure has two glass surfaces and a blackened surface at the bottom to ensure maximum capture of solar radiation.

The blackened surfaces increase the temperature of chamber air to reduce the temperature difference between the chamber and water tubes. At quasi-steady state, the temperatures of tube and enclosure become almost equal and result in negligible convection losses. Consider the following:

(1) $$\begin{array}{c} Q_{\text{loss}}=hA\Delta T\left(\text{minimum}\right). \end{array}$$

We have selected Ethylene-Propylene-Dimer M-Class synthetic rubber. EPDM rubber is easy to fabricate, noncorrosive, and cheaper. It is also not susceptible to scaling like metallic solar absorbers. Rubber compounds containing EPDM have better temperature resistance properties than ones containing natural rubber or styrene-butadiene rubber (SBR) [3]. Therefore, EPDM Rubber can withstand prolonged exposure to sunlight during different seasons without any deformation and degradation. EPDM Rubber can bear solar radiations for long time without any deterioration.

Metallic collectors have a serious problem of scaling (deposition of oxides on the inner surface of tubes due to oxidation of metal) in areas of hard water and fouling (deposition of insoluble salts and unwanted biomaterial due to aquatic environment). Such collectors have less resistivity to external corrosion even when coated. Corrosion effect also enhances due to the contact of dissimilar metals. When used in severe cold climates like in Quetta, Gilgit, Murree, and Kashmir collector is damaged as metals can crack or leak due to freezing temperatures during the night.

Analysis of the complete solar heater was under taken analytically in the following segments.

- Heating up characteristics of triangular chamber.
- Natural convection at inner surfaces of glass chambers.
- Conduction through glass thickness.
- Convection from outer surface of chamber glass.
- Heat absorption by EPDM tubes.

In order to analyze convection and conduction heat transfer phenomenon from the chamber, it is reasonable to model the chamber as an enclosure with a continuous heating from bottom and side plates. This assumption is valid because incident radiations are absorbed by the blackened bottom and side plates. In steady state, which is normally achieved within approximately 15 minutes (as discussed in later section), the bottom plate at a constant temperature will start radiating itself. This will increase the temperature of chamber to an arbitrary temperature depending upon the solar insolation. Increase in chamber temperature will reduce convection losses from the tube due to very small Δ*T*.

### 2.2. Mathematical Model

Assuming the system as the triangular enclosure with discrete heating from the bottom plate and natural convection taking place at surface of the plate. Natural convection heat transfer coefficient depends upon the Nusselt number. The Nusselt number can be calculated from Grashoff and Raleigh numbers as follows:

(2) $$\begin{array}{c} \text{Gr}=\frac{g\beta \left(T_{s}-T_{a}\right){L_{c}}^{3}}{U^{2}},\\ \text{Ra}=\text{Gr}\times \text{Pr}, \end{array}$$

where *g*: gravitational acceleration; *β*: coefficient of volume expansion, *β* = 1/*K* (*β* = 1/*T* for ideal gases); *T*_*s*_: temperature of the surface; *T*_*a*_: temperature of air sufficiently far from the surface; *L*_*c*_: characteristic length of geometry; *ν*: kinematic viscosity of fluid; Pr: Prandtl number.

Finally, the Nusselt number therefore can be found out by using the following formulas:

(3) $$\begin{array}{c} \text{Nu}=0.54\times {\left(\text{Ra}\right)}^{0.25}\;\text{for}\;\;10^{4}<\text{Ra}<10^{7},\\ \text{Nu}=0.15*{\left(\text{Ra}\right)}^{\left(1/3\right)}\;\text{for}\;\;10^{7}<\text{Ra}<10^{11}. \end{array}$$

Heat transfer coefficient is calculated by

(4) $$\begin{array}{c} h=\frac{\text{Nu}\times k}{x}, \end{array}$$

where *k* = thermal conductivity of fluid (i.e., air) and *x* = characteristic length.

#### 2.2.1. Natural Convection at Inner Surface of the Glass Chamber

Heat transfer coefficient for inner side convection is determined by the Nusselt number. The Nusselt number is taken from the experimental results available for the natural convection [4]. Convection coefficient is calculated [5] using (4), taking length of glass slab as characteristics length “*x.*”

#### 2.2.2. Conduction through Glass Thickness

The heat conducted by the glass slab can be found out by

(5) $$\begin{array}{c} Q=\frac{kA\left(T_{\text{gi}}-T_{\text{go}}\right)}{t}, \end{array}$$

where *k*: thermal conductivity; *A*: area of glass slab; *T*_gi_: inside glass surface temperature; *T*_go_: outside glass surface temperature.

#### 2.2.3. Convection from Outer Surface of Glass

At outer surface, forced convection is also expected due to wind speed approximately at 3 m/s. To ascertain the dominancy of forced convection Reynolds number (Re) is required as follows:

(6) Re=V×xν,

where Re: Reynolds number, *V*: wind speed (m/s), *x*: characteristic length of the glass slab, *ν*: kinematic viscosity of air.

As the natural and forced convections both exist in this case. So the dominance is explained by the factor [5] as follows:

(7) $$\begin{array}{c} \frac{\text{Gr}}{\text{R}{\text{e}}^{2}}. \end{array}$$

 If Gr/Re^2^ < 1, natural convection is dominant.
 If Gr/Re^2^ = 1, both natural and forced convection are to be taken in to account.
 If Gr/Re^2^ > 1, forced convection is dominant and is considered only.

By calculation in the new design, we found out that this ratio is less than one Gr/Re^2^ ≪ 1. Therefore, effects of natural convections are only considered here.

Now the Nusselt is calculated by [6] as follows:

(8) $$\begin{array}{c} {\text{Nu}}_{x}=\frac{hx_{x}}{k}=0.0296{\text{R}{\text{e}}_{x}}^{0.8}\text{P}{\text{r}}^{1/3}. \end{array}$$

Convection coefficient can then be calculated using (4).

#### 2.2.4. Time Required for Chamber Heating

**Figure 1:**
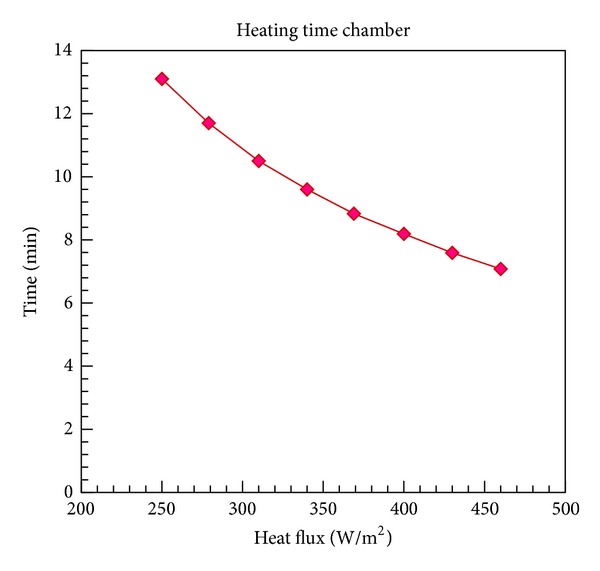
Heating time at different radiation rates.

Considering all losses and heat additions, we can calculate the heating time of the chamber. As it will be a function of incident radiation, Figure 1 shows that even at a minimum insolation, the heating time is not much.

#### 2.2.5. Heat Absorption by EPDM Tubes

Tube is considered to be exposed to a constant heat flux with inlet temperature equal to the ambient atmospheric temperature. System is being designed for an outlet temperature of 70°C. Keeping these constraints, the length of the tube is calculated as follows:

(9) $$\begin{array}{c} T_{e}=T_{i}+\frac{q_{s}A_{s}}{m\text{Cp}},\\ L=\frac{\left(T_{e}-T_{i}\right)m\text{Cp}}{2\pi rq_{s}}, \end{array}$$

where *T*_*e*_: exit temperature; *T*_*i*_: inlet temperature; *A*_*s*_: tube surface area; *L*: length of the tube; *m*: mass flow rate; Cp: specific heat capacity of fluid/water; *r*: radius of the tube; *q*_*s*_: heat transfer rate.

**Figure 2:**
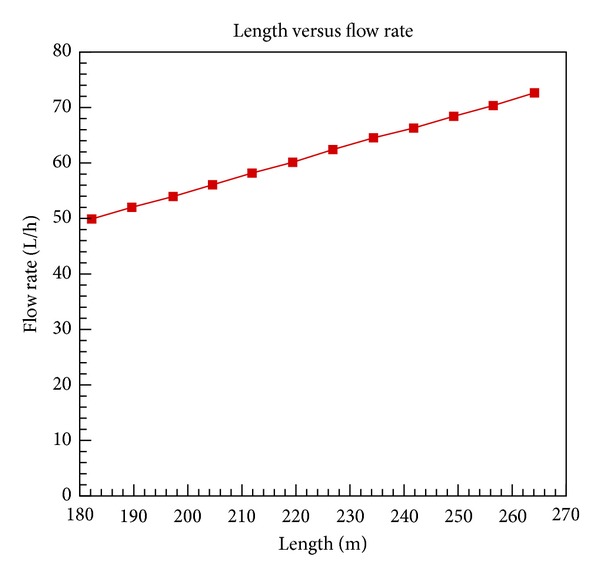
Flow rate and length relation at constant heat flux.

The calculations are performed at inlet and outlet temperatures of 25°C and 70°C, respectively, and a heat flux of 453 W/m^2^. The variation of tube length with flow rate is plotted in Figure 2. As the geyser is designed for the community housing so the required length for 450 liters per day (or 65 liters per hour) production is 800 ft (238 m) approximately as shown in Figure 2.

**Figure 3:**
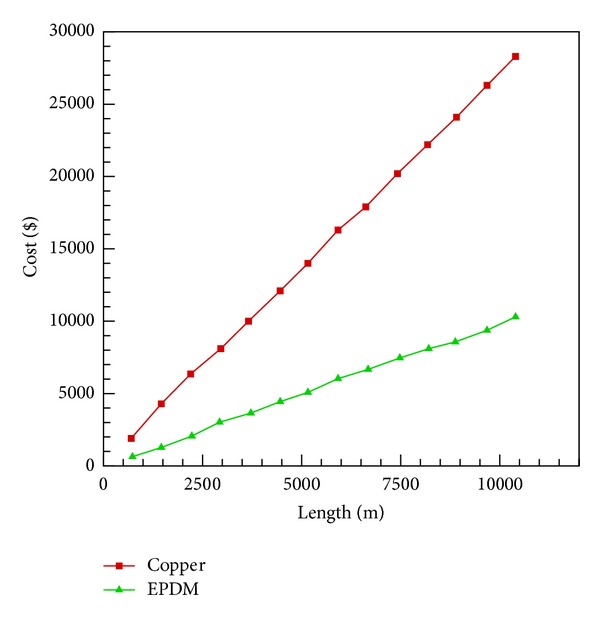
Cost comparison for EPDM and copper piping.

A cost analysis in USD was carried out between metallic copper and EPDM piping which is shown in Figure 3. Analysis is based upon the current market prices. It is also worth to mention that copper piping required additional coating of black nickel or black chrome to increase absorptive properties, whereas this additional treatment is not required in case of EPDM.

**Figure 4:**
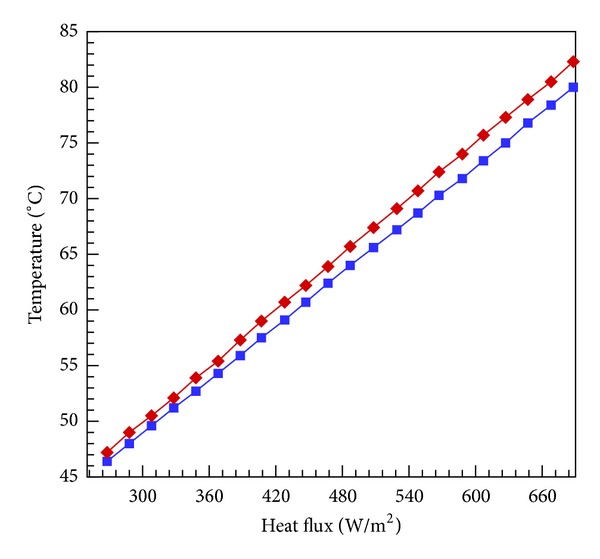
Temperature difference between inner and outer surface of the tube.

To further demonstrate the heat conduction capacity of EPDM rubber, a comparison is drawn between inner and outer surface temperature of the tubes.  Figure 4 shows the comparison between inner and outer surface temperature of the tube where we can see very small difference in both temperatures. The figure shows the difference at different heat fluxes.

### 2.3. Working of Heater in the Month of January

**Figure 5:**
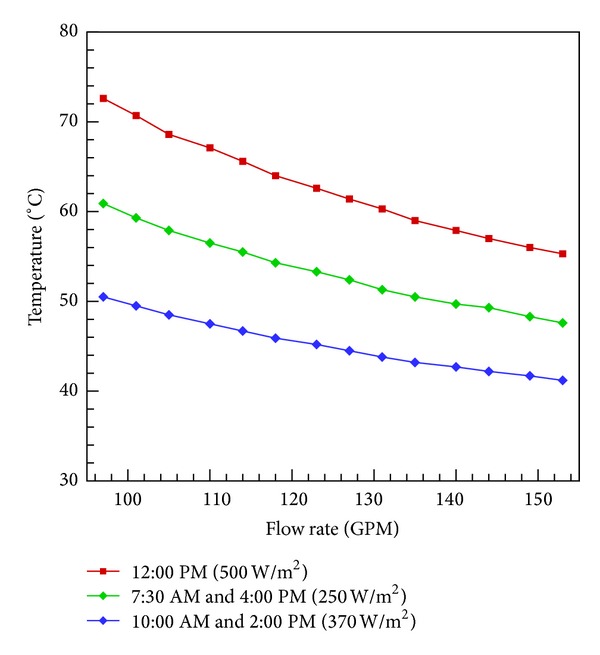
Performance analysis for the month of January.

Solar radiations are considered minimum in the month of January in the northern hemisphere. However, due to cold weather in these months, the need of domestic water heating is increased. Analytical formulations and calculations were carried out to check the working of the heater in the month of January.  Figure 5 shows its performance which is considered appropriate. Heat flux at different times was considered, it was found that even at 7:30 AM, when solar flux was 250 w/m^2^, the heater is able to produce heated water at temperature between 40 and 50°C.

### 2.4. Angle Simulation

One of the features of the heater is that glass is kept at an angle to ensure it can trap the solar radiation in early hours in morning and late hours in evening. These calculations are done by considering the effect of critical angle. The critical angle of glass varies from 38° to 43°. The angle of sun with the horizontal is (90 + solar hour angle) before solar noon and (180 − solar hour angle) after solar noon. The flint glass has the critical angle of 39°. Below this angle with the perpendicular of glass, all of the solar radiations will be reflected because of total internal reflection.

For flat glass, solar radiations up to an angle of 52° with the horizontal will be reflected. This equals the solar hour angle of 38° which means that the flat plate solar collector does not start working before 10 am in the morning and after 4 pm during the month of January. (Solar hour angles calculated for the latitude and longitude of 24 and 67 for Karachi [7]).

We simulated for different angles of enclosure slopes and we found that at 30° of glass inclination, we are getting maximum total radiations. This is because the heater starts to receive the solar radiations at 22° with the horizontal. At this instant, the solar hour angle is 68° and the starting time of geyser is thus 8 am in the morning. The end time will be 5 pm. These observations have been made for the month of January.

**Figure 6:**
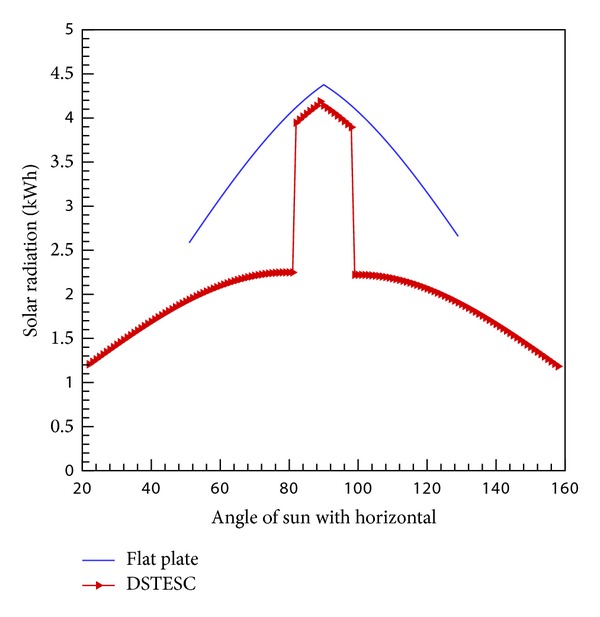
Comparison of radiation capture for flat and new concept.

The values of solar radiation intensity are taken at 40° south tilt for the month of January for Pakistan. Intensity varies throughout the day. We got the data at 12 points and interpolation is done for the points in between. Based on all the above factors, we have performed simulation for flat plate and novel design at different inclination angles. We concluded that inclination of 30° has shown the best results.  Figure 6 shows the comparison of flat plate with new design (30° glass inclination angle).

The sharp rise in the value of solar radiations absorbed increases when the angle of the sun with the horizontal rises above the 82° because at this instant both sides of glass enclosure start to pass radiation through them thus the area of glass to transmit the radiations is doubled. It is a basic phenomenon that as the area of the glass increases, more radiations will be transmitted through glass and will be absorbed on the black surface.

After performing the calculations, we found that the total radiation energy received by the collector on the basis of area of 6 m^2^ is 2262.482 KWh/day for flat plate and 2333.27 KWh/day for DSTESC. This gives an increased performance of approximately 3% for DSTESC.

## 3. Experimental Result

**Figure 7:**
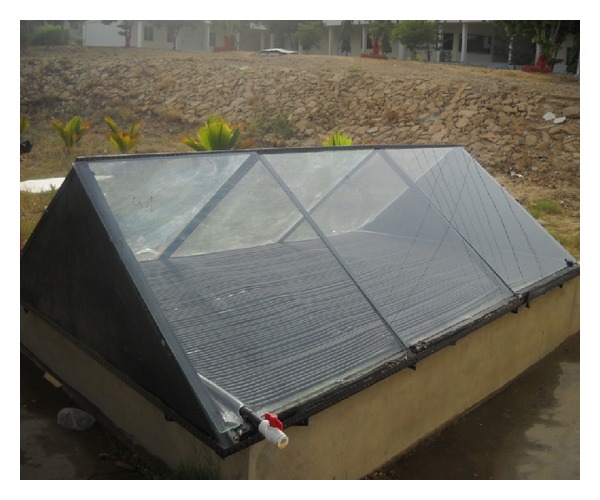
Final assembly of DSTEC.

Solar heater named as DSTESC was fabricated in the lab as per designed specification. It was important to ensure that base plate is properly insulated from underneath to decrease conduction losses from blackened bottom plate. Expanded polystyrene (EPS) foam is considered an excellent insulator for such application. EPS has thermal conductivity of 0.03 W/m·K at nominal density between 25 g/L and 35 g/L [8]. A three-inch slab of EPS foam supported by wooden base is installed under bottom plate.  Figure 7 shows the complete solar heater used for experimentation.

**Figure 8:**
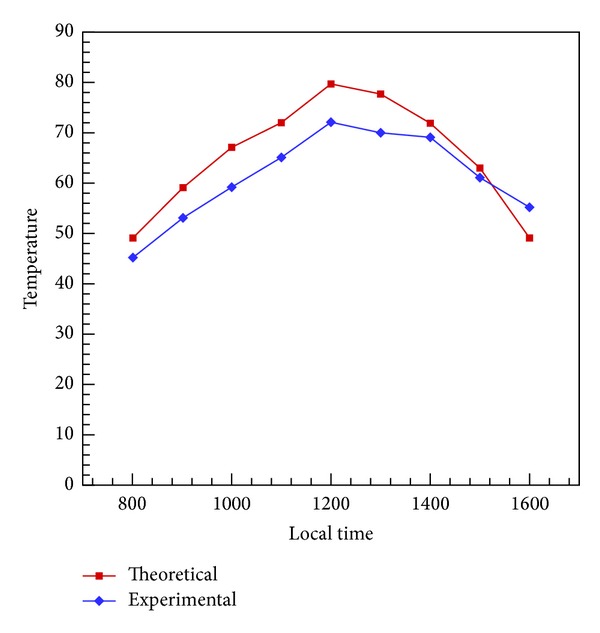
Comparison of analytical and experimental results.

In experimentation phase, performance of DSTEC was tested without top glass plate. Such arrangements are required for large volume of water heating like the swimming pool. Temperature achieved was between 44°C and 46°C. This is considered sufficient for the swimming pool water heating. We tested the system at 16:00 hours to 17:00 hours (GMT+5). From this we concluded that if we use this system without glass and with relatively simple base, we can easily perform swimming pool water heating. In the second stage, 5 mm thick glass was mounted on the heater to produce green house effect and reduce convection losses. The inclination angle of glass slab after performing simulations was optimized at 30° from the horizontal. This angle will ensure that sun rays will never exceed critical angle of glass, hence reducing reflection losses to a minimum. Comparison of analytical and experimental results is shown in Figure 8.

**Figure 9:**
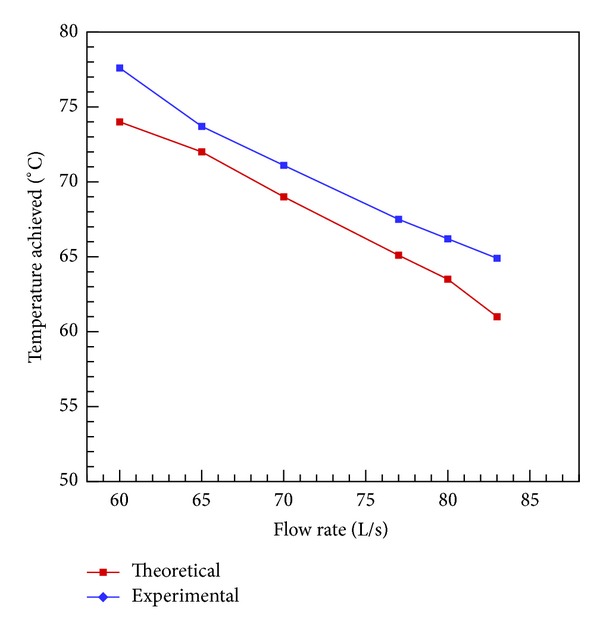
Flow rate versus outlet temperature.

Effect on temperature was also compared with increasing flow rate to optimize the output of the heater.  Figure 9 shows a good agreement to analytical results. Based on these results, it is appropriate to run the system for a capacity of 60 liters/hour.

## 4. Conclusion and Future Work

The novel approach described in the paper is considered more cost effective as compared to evacuated tube or flat plate metallic tube water heaters. The performance of double slope arrangement is more by 3% as compared to flat plate configuration. As domestic water heating can be met easily by a temperature of 70°C, current system can produce approximately 540 liters in a day which is considered enough to meet the requirement. Experimental and analytical results are found in good agreement. Numerical simulation of the novel design will be carried out as future work to bring out a more optimized design.
